# Defining the causes of sporadic Parkinson’s disease in the global Parkinson’s genetics program (GP2)

**DOI:** 10.1038/s41531-023-00533-w

**Published:** 2023-09-12

**Authors:** Clodagh Towns, Madeleine Richer, Simona Jasaityte, Eleanor J. Stafford, Julie Joubert, Tarek Antar, Alejandro Martinez-Carrasco, Mary B. Makarious, Bradford Casey, Dan Vitale, Kristin Levine, Hampton Leonard, Caroline B. Pantazis, Laurel A. Screven, Dena G. Hernandez, Claire E. Wegel, Justin Solle, Mike A. Nalls, Cornelis Blauwendraat, Andrew B. Singleton, Manuela M. X. Tan, Hirotaka Iwaki, Huw R. Morris, the Global Parkinson’s Genetics Program (GP2), Emilia M. Gatto, Marcelo Kauffman, Samson Khachatryan, Zaruhi Tavadyan, Claire E. Shepherd, Julie Hunter, Kishore Kumar, Melina Ellis, Miguel E. Rentería, Sulev Koks, Alexander Zimprich, Artur F. Schumacher-Schuh, Carlos Rieder, Paula Saffie Awad, Vitor Tumas, Sarah Camargos, Edward A. Fon, Oury Monchi, Ted Fon, Benjamin Pizarro Galleguillos, Marcelo Miranda, Maria Leonor Bustamante, Patricio Olguin, Pedro Chana, Beisha Tang, Huifang Shang, Jifeng Guo, Piu Chan, Wei Luo, Gonzalo Arboleda, Jorge Orozco, Marlene Jimenez del Rio, Alvaro Hernandez, Mohamed Salama, Walaa A. Kamel, Yared Z. Zewde, Alexis Brice, Jean-Christophe Corvol, Ana Westenberger, Anastasia Illarionova, Brit Mollenhauer, Christine Klein, Eva-Juliane Vollstedt, Franziska Hopfner, Günter Höglinger, Harutyun Madoev, Joanne Trinh, Johanna Junker, Katja Lohmann, Lara M. Lange, Manu Sharma, Sergiu Groppa, Thomas Gasser, Zih-Hua Fang, Albert Akpalu, Georgia Xiromerisiou, Georgios Hadjigorgiou, Ioannis Dagklis, Ioannis Tarnanas, Leonidas Stefanis, Maria Stamelou, Efthymios Dadiotis, Alex Medina, Germaine Hiu-Fai Chan, Nancy Ip, Nelson Yuk-Fai Cheung, Phillip Chan, Xiaopu Zhou, Asha Kishore, Divya KP, Pramod Pal, Prashanth Lingappa Kukkle, Roopa Rajan, Rupam Borgohain, Mehri Salari, Andrea Quattrone, Enza Maria Valente, Lucilla Parnetti, Micol Avenali, Tommaso Schirinzi, Manabu Funayama, Nobutaka Hattori, Tomotaka Shiraishi, Altynay Karimova, Gulnaz Kaishibayeva, Cholpon Shambetova, Rejko Krüger, Ai Huey Tan, Azlina Ahmad-Annuar, Mohamed Ibrahim Norlinah, Nor Azian Abdul Murad, Shahrul Azmin, Shen-Yang Lim, Wael Mohamed, Yi Wen Tay, Daniel Martinez-Ramirez, Mayela Rodriguez-Violante, Paula Reyes-Pérez, Bayasgalan Tserensodnom, Rajeev Ojha, Tim J. Anderson, Toni L. Pitcher, Arinola Sanyaolu, Njideka Okubadejo, Oluwadamilola Ojo, Jan O. Aasly, Lasse Pihlstrøm, Manuela Tan, Shoaib Ur-Rehman, Mario Cornejo-Olivas, Maria Leila Doquenia, Raymond Rosales, Angel Vinuela, Elena Iakovenko, Bashayer Al Mubarak, Muhammad Umair, Eng-King Tan, Jia Nee Foo, Ferzana Amod, Jonathan Carr, Soraya Bardien, Beomseok Jeon, Yun Joong Kim, Esther Cubo, Ignacio Alvarez, Janet Hoenicka, Katrin Beyer, Maria Teresa Periñan, Pau Pastor, Sarah El-Sadig, Christiane Zweier, Paul Krack, Chin-Hsien Lin, Hsiu-Chuan Wu, Pin-Jui Kung, Ruey-Meei Wu, Yihru Wu, Rim Amouri, Samia Ben Sassi, A. Nazlı Başak, Gencer Genc, Özgür Öztop Çakmak, Sibel Ertan, Alastair Noyce, Anette Schrag, Anthony Schapira, Camille Carroll, Claire Bale, Donald Grosset, Henry Houlden, John Hardy, Kin Ying Mok, Mie Rizig, Nicholas Wood, Nigel Williams, Olaitan Okunoye, Patrick Alfryn Lewis, Rauan Kaiyrzhanov, Rimona Weil, Seth Love, Simon Stott, Simona Jasaitye, Sumit Dey, Vida Obese, Alberto Espay, Alyssa O’Grady, Andrew K. Sobering, Bernadette Siddiqi, Brian Fiske, Cabell Jonas, Carlos Cruchaga, Charisse Comart, Claire Wegel, Deborah Hall, Dena Hernandez, Ejaz Shiamim, Ekemini Riley, Faraz Faghri, Geidy E. Serrano, Honglei Chen, Ignacio F. Mata, Ignacio Juan Keller Sarmiento, Jared Williamson, Jonggeol Jeff Kim, Joseph Jankovic, Joshua Shulman, Justin C. Solle, Kaileigh Murphy, Karen Nuytemans, Karl Kieburtz, Katerina Markopoulou, Kenneth Marek, Kristin S. Levine, Lana M. Chahine, Laura Ibanez, Laurel Screven, Lauren Ruffrage, Lisa Shulman, Luca Marsili, Maggie Kuhl, Marissa Dean, Mathew Koretsky, Megan J. Puckelwartz, Miguel Inca-Martinez, Naomi Louie, Niccolò Emanuele Mencacci, Roger Albin, Roy Alcalay, Ruth Walker, Sara Bandres-Ciga, Sohini Chowdhury, Sonya Dumanis, Steven Lubbe, Tao Xie, Tatiana Foroud, Thomas Beach, Todd Sherer, Yeajin Song, Duan Nguyen, Toan Nguyen, Masharip Atadzhanov

**Affiliations:** 1 Department of Clinical and Movement Neurosciences, Queen Square Institute of Neurology, University College London, London, UK; 2 University College London, London, UK; 3 Molecular Genetics Section, Laboratory of Neurogenetics, National Institute on Aging, National Institutes of Health, Bethesda, MD USA; 4 National Institutes of Health, Bethesda, MD USA; 5 Department of Clinical Research, Michael J. Fox Foundation for Parkinson’s Research, New York City, NY USA; 6 The Michael J. Fox Foundation for Parkinson’s Research, New York, NY USA; 7 Center for Alzheimer’s and Related Dementias (CARD), National Institute on Aging and National Institute of Neurological Disorders and Stroke, National Institutes of Health, Bethesda, MD USA; 8 Data Tecnica International, Washington, DC USA; 9 National Institute on Aging/National Institutes of Health, Bethesda, MD USA; 10 Department of Medical and Molecular Genetics, Indiana University School of Medicine, Indianapolis, IN USA; 11 Integrative Genomics Unit, Laboratory of Neurogenetics, National Institute on Aging, National Institutes of Health, Bethesda, MD USA; 12 National Institute on Aging, Bethesda, MD USA; 13 Department of Neurology, Oslo University Hospital, Oslo, Norway; 14 Sanatorio de la Trinidad Mitre- INEBA, Buenos Aires, Argentina; 15 Hospital JM Ramos Mejia, Buenos Aires, Argentina; 16 Somnus Neurology Clinic, Yerevan, Armenia; 17 Neuroscience Research Australia, Sydney, NSW Australia; 18 ANZAC Research Institute, Concord, NSW Australia; 19 Garvan Institute of Medical Research and Concord Repatriation General Hospital, Darlinghurst, NSW Australia; 20 Concord Hospital, Concord, NSW Australia; 21 QIMR Berghofer Medical Research Institute, Herston, QLD Australia; 22 Murdoch University, Perth, Australia; 23 Medical University Vienna Austria, Vienna, Austria; 24 Universidade Federal do Rio Grande do Sul / Hospital de Clínicas de Porto Alegre, Porto Alegre, Brazil; 25 Federal University of Health Sciences of Porto Alegre, Porto Alegre, Brazil; 26 Universidade Federal do Rio Grande do Sul, Porto Alegre, Brazil; 27 University of São Paulo, São Paulo, Brazil; 28 Universidade Federal de Minas Gerais, Belo Horizonte, Brazil; 29 Montreal Neurological Institute, Montreal, QC Canada; 30 Institut universitaire de gériatrie de Montréal, Montreal, QC Canada; 31 McGill University, Montreal, QC Canada; 32 Universidad de Chile, Santiago, Chile; 33 Fundación Diagnosis, Santiago, Chile; 34 Faculty of Medicine Universidad de Chile, Santiago, Chile; 35 CETRAM, Santiago, Chile; 36 Central South University, Changsha, China; 37 West China Hospital Sichuan University, Chengdu, China; 38 Xiangya Hospital, Changsha, China; 39 Capital Medical University, Beijing, China; 40 Zhejiang University, Hangzhou, China; 41 Universidad Nacional de Colombia, Bogotá, Colombia; 42 Fundación Valle del Lili, Santiago De Cali, Colombia; 43 University of Antioquia, Medellin, Colombia; 44 University of Costa Rica, San Jose, Costa Rica; 45 The American University in Cairo, Cairo, Egypt; 46 Beni-Suef University, Beni Suef, Egypt; 47 Addis Ababa University, Addis Ababa, Ethiopia; 48 Paris Brain Institute, Paris, France; 49 Sorbonne Université, Paris, France; 50 University of Lübeck, Lübeck, Germany; 51 Deutsches Zentrum für Neurodegenerative Erkrankungen, Göttingen, Germany; 52 University Medical Center Göttingen, Göttingen, Germany; 53 Department of Neurology, University Hospital, LMU Munich, Munich, Germany; 54 University of Lübeck and University Medical Center Schleswig-Holstein, Lübeck, Germany; 55 University of Tubingen, Tübingen, Germany; 56 University of Mainz, Mainz, Germany; 57 The German Center for Neurodegenerative Diseases, Göttingen, Germany; 58 University of Ghana Medical School, Accra, Ghana; 59 University of Thessaly, Volos, Greece; 60 Aristotle University of Thessaloniki, Thessaloniki, Greece; 61 Ionian University, Corfu, Greece; 62 Biomedical research Foundation of the Academy of Athens, Athens, Greece; 63 Diagnostic and Therapeutic Centre HYGEIA Hospital, Marousi, Greece; 64 Hospital San Felipe, Tegucigalpa, Honduras; 65 Queen Elizabeth Hospital, Kowloon, Hong Kong; 66 The Hong Kong University of Science and Technology, Kowloon, Hong Kong; 67 Aster Medcity, Kochi, India; 68 Sree Chitra Tirunal Institute for Medical Sciences and Technology, Thiruvananthapuram, India; 69 National Institute of Mental Health & Neurosciences, Bengaluru, India; 70 Manipal Hospital, Delhi, India; 71 All India Institute of Medical Sciences, Delhi, India; 72 Nizam’s Institute Of Medical Sciences, Hyderabad, India; 73 Shahid Beheshti University of Medical Science, Tehran, Iran; 74 Magna Græcia University of Catanzaro, Catanzaro, Italy; 75 University of Pavia, Pavia, Italy; 76 University of Perugia, Perugia, Italy; 77 University of Rome Tor Vergata, Rome, Italy; 78 Juntendo University, Tokyo, Japan; 79 Juntendo University faculty of medicine, Tokyo, Japan; 80 Jikei University School of Medicine, Tokyo, Japan; 81 Institute of Neurology and Neurorehabilitation, Almaty, Kazakhstan; 82 Kyrgyz State Medical Academy, Bishkek, Kyrgyzstan; 83 University of Luxembourg, Luxembourg, Luxembourg; 84 University of Malaya, Kuala Lumpur, Malaysia; 85 Universiti Kebangsaan Malaysia, Selangor, Malaysia; 86 UKM Medical Molecular Biology Institute, Kuala Lumpur, Malaysia; 87 Universiti Kebangsaan Malaysia Medical Centre, Kuala Lumpur, Malaysia; 88 International Islamic University, Kuala Lumpur, Malaysia; 89 Tecnologico de Monterrey, Monterrey, Mexico; 90 Instituto Nacional de Neurologia y Neurocirugia, Mexico City, Mexico; 91 Universidad Nacional Autónoma de México, Mexico City, Mexico; 92 Mongolian National University of Medical Sciences, Ulaanbaatar, Mongolia; 93 Tribhuvan University, Kirtipur, Nepal; 94 University of Otago, Dunedin, New Zealand; 95 University of Lagos, Lagos, Nigeria; 96 College of Medicine of the University of Lagos, Lagos, Nigeria; 97 Norwegian University of Science and Technology, Trondheim, Norway; 98 Oslo University Hospital, Oslo, Norway; 99 University of Science and Technology Bannu, Bannu, Pakistan; 100 Universidad Cientifica del Sur, Lima, Peru; 101 Metropolitan Medical Center, Manila, Philippines; 102 University of Puerto Rico, San Juan, Puerto Rico; 103 Research Center of Neurology, Moscow, Russia; 104 King Faisal Specialist Hospital and Research Center, Riyadh, Saudi Arabia; 105 King Abdullah International Medical Research Center, Jeddah, Saudi Arabia; 106 National Neuroscience Institute, Singapore, Singapore; 107 Nanyang Technological University, Singapore, Singapore; 108 University of KwaZulu-Natal, Durban, South Africa; 109 University of Stellenbosch, Stellenbosch, South Africa; 110 Stellenbosch University, Stellenbosch, South Africa; 111 Seoul National University Hospital, Seoul, South Korea; 112 Yongin Severance Hospital, Seoul, South Korea; 113 Hospital Universitario Burgos, Burgos, Spain; 114 University Hospital Mutua Terrassa, Barcelona, Spain; 115 Institut de Recerca Sant Joan de Deu, Barcelona, Spain; 116 Research Institute Germans Trias i Pujol, Barcelona, Spain; 117 Instituto de Biomedicina de Sevilla, Seville, Spain; 118 University Hospital Germans Trias i Pujol, Barcelona, Spain; 119 Faculty of medicine university of Khartoum, Khartoum, Sudan; 120 Inselspital Bern, University of Bern, Bern, Switzerland; 121 National Taiwan University Hospital, Taipei City, Taiwan; 122 Chang Gung Memorial Hospital, Taoyuan City, Taiwan; 123 National Taiwan University, Taipei City, Taiwan; 124 National Institute Mongi Ben Hamida of Neurology, Tunis, Tunisia; 125 Mongi Ben Hmida National Institute of Neurology, Tunis, Tunisia; 126 Koç University, Istanbul, Turkey; 127 Sisli Etfal Training and Research Hospital, Istanbul, Turkey; 128 Queen Mary University of London, London, UK; 129 University of Plymouth, Plymouth, UK; 130 Parkinson’s UK, London, UK; 131 University of Glasgow, Glasgow, UK; 132 Cardiff University, Cardiff, UK; 133 Royal Veterinary College University of London, London, UK; 134 University of Bristol, Bristol, UK; 135 Cure Parkinson’s, London, UK; 136 University of Cincinnati, Cincinnati, OH USA; 137 Augusta University / University of Georgia Medical Partnership, Augusta, GA USA; 138 Mid-Atlantic Permanente Medical Group, Bethesda, MD USA; 139 Washington University, St. Louis, MO USA; 140 Indiana University, Bloomington, IN USA; 141 Rush University, Chicago, IL USA; 142 Kaiser Permanente, Oakland, CA USA; 143 Coalition for Aligning Science, Washington, WA USA; 144 Banner Sun Health Research Institute, Sun City, AZ USA; 145 Michigan State University, East Lansing, MI USA; 146 Cleveland Clinic, Cleveland, OH USA; 147 Northwestern University, Evanston, IL USA; 148 Baylor College of Medicine, Houston, TX USA; 149 Baylor College of Medicine, Texas Children’s Hospital, Houston, TX USA; 150 University of Miami Miller School of Medicine, Miami, FL USA; 151 Beth Israel Deaconess Medical Center, Boston, MA USA; 152 North Shore University Health System, Chicago, IL USA; 153 Institute for Neurodegenerative Disorders, New Haven, CT USA; 154 University of Pittsburgh, Pittsburgh, PA USA; 155 University of Alabama at Birmingham, Birmingham, AL USA; 156 University of Maryland, Baltimore, MD USA; 157 Northwestern University, Chicago, IL USA; 158 Universit of Michigan, Ann Arbor, MI USA; 159 Columbia University, New York, NY USA; 160 James J. Peters Veterans Affairs Medical Center, New York, NY USA; 161 Aligning Science Across Parkinson’s, Washington, WA USA; 162 University of Chicago, Chicago, IL USA; 163 Indiana University School of Medicine, Indianapolis, IN USA; 164 Sun Health Research Institution, Sun City, AZ USA; 165 Hue University, Huế, Vietnam; 166 University of Zambia, Lusaka, Zambia

**Keywords:** Parkinson's disease, Parkinson's disease, Parkinson's disease, Clinical genetics, Cognitive ageing

## Abstract

The Global Parkinson’s Genetics Program (GP2) will genotype over 150,000 participants from around the world, and integrate genetic and clinical data for use in large-scale analyses to dramatically expand our understanding of the genetic architecture of PD. This report details the workflow for cohort integration into the complex arm of GP2, and together with our outline of the monogenic hub in a companion paper, provides a generalizable blueprint for establishing large scale collaborative research consortia.

## Introduction

Parkinson’s disease (PD) is a multifactorial disorder with complex etiology. The largest genome-wide association study (GWAS) to-date included 37,688 cases, 18,618 proxy cases (unaffected first-degree relatives), and 1.4 million controls from European ancestry, and identified 90 independent risk signals across 78 genomic regions; 38 of which were novel signals^1^. Despite these advances, PD GWAS are currently limited by scale, a focus on European populations, and limited integration with clinical phenotype data.

A power calculation based on the 2019 GWAS data indicates that inclusion of an additional ~99,000 cases would enable variants of smaller effect size that contribute to polygenic risk (*p*-value cut off: 1.35 × 10^−3^) to reach genome-wide significance. Therefore, expanding PD GWAS to at least this size will result in identification of additional risk loci and improve genetic prediction of PD occurrence^1^. The heritability of PD can be estimated using twin studies or statistical genetic methods and is thought to lie between 22% and 40% in European populations. Known genome wide significant loci currently explain ~16% of the heritability of PD^1^. The use of polygenic risk score analysis (including loci that do not reach genome wide significance) indicates that there are likely to be a substantial number of loci contributing to PD risk that have not yet been defined. Our power analysis indicates that 99,000 PD cases will be needed to define loci with 80% power, a minor allele frequency of 0.21 and similar effects to the current state-of-the-art analysis. The variability of phenotypes observed in PD are likely to have a genetic basis^2–4^. Knowledge of associations between genotype and clinical outcomes will enable clinicians to provide patients with a more accurate prognosis. Understanding the gene-to-phenotype pathways responsible for specific PD features would provide an opportunity to develop treatments targeting phenotype-specific disease pathways, resulting in more efficient and personalized treatment with fewer side effects. We aim to capture the diversity of PD outcomes, including Parkinson’s itself but also related conditions such as prodromal Parkinson’s, dementia with Lewy bodies and other Parkinson’s plus syndromes, and to perform large-scale analyses of clinical-genetic data with sufficient power for gene discovery. This will comprise regression and time-to-event analysis for the phenotype of interest (e.g., dementia, dyskinesias, motor progression). It is likely that this will be limited to around 25% of samples included in this study with in depth longitudinal data, and further large scale longitudinal cohorts will be needed to explore the biology of progression and diverse phenotypes. The focus of PD GWAS on individuals of European ancestry has left gaps in our knowledge of PD-associated genetic variants in underrepresented populations and limited our ability to resolve GWAS loci^5^. To advance understanding of the genetic determinants of Parkinsonism on a global scale, we need to ensure representation of diverse ancestries with sample sizes sufficient to detect ancestry-specific signals. We aim to include at least 15,000 participants of African, South Asian, and East Asian ancestry, respectively.

The Global Parkinson’s Genetics Program (GP2, http://gp2.org/), funded by the Aligning Science Across Parkinson’s initiative (ASAP, https://parkinsonsroadmap.org/) will recruit PD, Prodromal Parkinson’s and Parkinson’s Plus (including Dementia with Lewy bodies, Progressive Supranuclear Palsy, Multiple System Atrophy and Cortico-basal syndrome) cohorts from across the world^6^. We will genotype >150,000 participants and integrate genetic data with harmonized clinical data for use in case-control and genotype-phenotype association studies. This figure will include a minimum of 50,000 individuals from ancestries currently underrepresented in Parkinson’s research: including Black American, African, Middle Eastern, Central/ East/South Asian, Indian, Caribbean and Central/South American. The remaining participants are expected to be of European ancestry^6^. Cleaned genetic and clinical data will be harmonized across cohorts and made available to Parkinson’s, Prodromal and Parkinson’s Plus researchers via a controlled-access online repository with a unified user agreement. Contributing investigators are encouraged to play an active role in the project by proposing and leading analyses, and will receive authorship on GP2 publications as well as support for their analyses. GP2 also provides comprehensive training opportunities for researchers from contributing institutions via an online learning management system. The outcome of GP2 will be an open access resource which will integrate clinical and genetic data from a very large, diverse sample of cases, and facilitate discovery of new genetic determinants of Parkinson’s, Prodromal and Parkinson’s Plus occurrence and phenotypic variation in multiple ancestries. A global network of PD researchers will be established, facilitating future collaboration and advancement of PD research. The project began in January 2020 and current funding extends to 2029.

Coordinating cohorts around the world to include 150,000 participants is a considerable logistical challenge. As of May 2022, we have established a workflow for cohort integration, a standardized set of clinical data elements, a recommended consent template, a protocol for evaluating cohorts joining the study, and a process for integrating and harmonizing the incoming clinical data. More broadly, we have established the complex hub framework, which created a code of conduct and publication policy, created a DNA quality and shipping protocol, and created the NeuroBooster Array (NBA) genotyping workflow.

The size and depth of the dataset which is being generated by GP2 will provide a major opportunity to discover new genotype-phenotype associations. Core analyses, analyses that will be continuously updated by the analysis teams at GP2 with the inclusion of new samples, will include case-control, age at onset, and progression GWAS, as well as within-ancestry analyses of previously underrepresented populations. Beyond this, GP2 is supporting additional analytical projects proposed by contributing collaborators. The GP2 network will also facilitate collaboration on auxiliary studies between investigators sharing the same area of interest (e.g., biomarkers of different modalities) to address outstanding questions in PD research.

GP2 is conducted according to overarching principles of democratization of data, collaboration and cooperation, safe and responsible data sharing, commitment to diversity in research, transparency and reproducibility, and production of an actionable resource in accordance with the Findable, Accessible, Interoperable and Reusable (FAIR) scientific data management principles^7^. It is GP2 policy that local researchers are included in publications that use their data/samples. Here, we report the specific steps undertaken by the Cohort Integration Working Group (CIWG) to identify, recruit, and harmonize clinical cohorts within the complex disease arm of GP2. GP2 also has a monogenic arm (https://gp2.org/working-groups/) which focuses on cases with potential monogenic causes of PD, i.e., those with early age at onset or a family history of PD (Reference: PMID:37369645).

## Methods

PD and Parkinson’s Plus investigators and cohorts are identified through relevant publications, or through existing consortia. We welcome contact from interested investigators around the world (Email: cohort@gp2.org). Prospective collaborators complete a Site Interest Form (SIF) (Supplementary Material) to provide a brief overview of their cohort [Supplementary material]. Data availability, type of cohort (e.g., brain bank, drug study etc.), ethnic diversity and sample/data sharing restrictions are all considered by the CIWG when reviewing SIF submissions. Cohorts’ consent documents are reviewed by the Operations and Compliance Working Group (OCWG) to ensure consent for sample/data sharing was obtained, and compliance with regional and cohort-specific data sharing restrictions. The OCWG assists the investigator if revisions are needed, and template consent language is provided online (https://gp2.org/resources/consent-guidelines/ [Supplementary Material]). We have an inclusive approach; to-date >95% of cohorts evaluated have been accepted. Following cohort approval, a concise Collaboration Agreement is signed by the collaborating PI and the Michael J. Fox Foundation (MJFF), and the necessary material and data transfer agreements are completed. The cohort’s samples are then transferred to one of GP2’s genotyping centers, and data are transferred through secure upload to a cloud server.

Once the cohort’s clinical data have been transferred, harmonization and quality control (QC) are performed by the CIWG, following a standard framework^8^. Core clinical data were decided on the basis of clinical scales available in existing PD cohorts (primarily the Tracking Parkinson’s^9^, PPMI^10^, PDBP^11^, NET PD-LS1^12^, and GEoPD^13^ cohorts) as well as on the basis of recent proposals for a modular set of assessment criteria to characterize longitudinal PD cohorts^14,15^. The core clinical data elements are the clinical data categories of interest which will be harmonized across cohorts and used for analysis (Table 1). We have generated a common dataset for brain banks, and have added data elements for time-to-event analysis (Supplementary Table 1). We are also collecting information on availability of other data and samples, including, plasma, serum, RNA, fibroblasts, CSF, skin biopsy, PBL, iPS to expand awareness of the resources that are available at contributing sites (Supplementary Material). The common dataset is defined in a common data dictionary (Supplementary Table 2). The harmonization of raw data uses a set of coding rules for re-coding and handling of missing data, using a modified custom script for each cohort.

**Table 1:** Recommended core clinical data.

	MINIMUM	MINIMUM PLUS	CORE	EXTENDED
Demographics (e.g., NINDS CDE General Core)^16^	✓	✓	✓	✓
Recruitment category (Case/Control)	✓	✓	✓	✓
Family History	✓	✓	✓	✓
Diagnostic checklist (MDS clinical diagnosis criteria^17^ or UK Parkinson’s Disease Society Brain Bank Diagnostic Criteria)^18^		✓	✓	✓
Primary diagnosis and PD certainty (PPMI Primary Clinical Diagnosis)^10^		✓	✓	✓
PD history (e.g., NINDS CDE for Parkinson’s Disease)^16^		✓	✓	✓
Global PD severity (CISI-PD)^19^		✓	✓	✓
Behavioral and Environmental History (PD-RFQ-U)^16^			✓	✓
Medical History			✓	✓
Current Medication Status			✓	✓
nM-EDL (MDS-UPDRS Part I [or UPDRS])^20,21^			✓	✓
M-EDL (MDS-UPDRS Part II [or UPDRS])^20,21^			✓	✓
Motor Assessment (MDS-UPDRS Part III [or UPDRS])^20,21^			✓	✓
Complications (MDS-UPDRS Part IV [or UPDRS])^20,21^			✓	✓
Cognitive Assessment (MMSE, MoCA)^22,23^			✓	✓
Motor (Hoehn and Yahr stage)^24^			✓	✓
Autonomic function assessment (SCOPA-AUT)^25^			✓	✓
pRBD (RBDSQ)^26^			✓	✓
Day time sleepiness (ESS)^27^				✓
Depression (GDS-15)^28^				✓
Orthostatic hypotension (Standing/sitting/supine HR and BP)				✓
Olfactory function (UPSIT or Sniffin-stick or BSIT)^29–31^				✓
General ADL (Schwab & England ADL)^32^				✓
PD EQL (PDQ-8)^33^				✓
Pain (Kings PD Pain Scale)^34^				✓

**Fig. 1:**
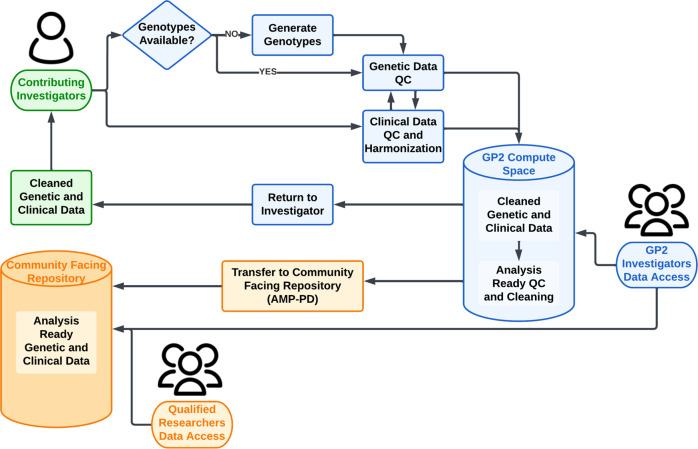
Cohort integration workflow. Data are contributed and returned to the local PI (green); new genetic data, data cleaning and harmonization are carried out by GP2 and made available for analysis within the GP2 consortium (blue); and data are released to qualified investigators via AMP-PD (orange). AMP-PD Accelerating Medicines Partnership - Parkinson’s Disease (https://amp-pd.org).

DNA samples from each cohort are genotyped on the Neuro Booster Array (NBA; https://github.com/GP2code/Neuro_Booster_Array), developed in collaboration between Data Tecnica International, NIA, NINDS, Illumina Inc and GP2. The NBA consists of the Illumina Infinium Global Diversity Array (GDA; https://www.illumina.com/products/by-type/microarray-kits/infinium-global-diversity.html), a high-density (1.9 M total variants) global backbone optimized for cross-population imputation coverage of the human genome, and additional custom content (>95,000 variants) which includes known causal variants for various neurodegenerative diseases and imputation boosters for underrepresented populations. QC of genotype data by the Data Analysis Working Group (DAWG) follows a standard pipeline. A custom genotype clustering file is used to ensure representation of the diverse genetic ancestries within the GP2 data. The clustering file used for the latest data release is based on 2793 samples across 6 ancestry groups, and includes 420 Gaucher disease cases to capture variants of interest in the *GBA1* risk gene. This file is available for download via the GP2 Github repository (https://github.com/GP2code). Cleaned genetic data are returned to the contributing investigator to use as they wish, and are uploaded to the GP2 repository for use in combined analyses (Fig. 1). GP2 covers the costs of genotyping and sample shipment for all contributing cohorts, and can assist investigators with the analysis of their cohort’s data.

The dataset which we are currently aggregating and harmonizing contains a wide variety of demographic and clinical factors, thanks to the diversity of contributing cohorts. As of March 2023, the CIWG has approved 145 cohorts for inclusion in GP2, of which 128 have been approved by the OCWG, and 74 have completed all necessary agreements and are in the process of transferring samples and clinical data. Samples that have been transferred are currently being genotyped and passed through the data QC pipeline. So far, the approved cohorts span over 50 different countries and territories. A map showing the geographic distribution of these cohorts, as well as current expected and completed sample numbers, can be found on the GP2 website (https://gp2.org/cohort-dashboard/ [Supplementary Material]).

### Reporting summary

Further information on research design is available in the Nature Research Reporting Summary linked to this article.

### Supplementary information

Global Parkinson’s Genetics Program: Emilia M Gatto, Marcelo Kauffman, Samson Khachatryan, Zaruhi Tavadyan, Claire E Shepherd, Julie Hunter, Kishore Kumar, Melina Ellis, Miguel E. Rentería, Sulev Koks, Alexander Zimprich, Artur F. Schumacher-Schuh, Carlos Rieder, Paula Saffie Awad, Vitor Tumas, Sarah Camargos, Edward A. Fon, Oury Monchi, Ted Fon, Benjamin Pizarro Galleguillos, Marcelo Miranda, Maria Leonor Bustamante, Patricio Olguin, Pedro Chana, Beisha Tang, Huifang Shang, Jifeng Guo, Piu Chan, Wei Luo, Gonzalo Arboleda, Jorge Orozco, Marlene Jimenez del Rio, Alvaro Hernandez, Mohamed Salama, Walaa A. Kamel, Yared Z. Zewde, Alexis Brice, Jean-Christophe Corvol, Ana Westenberger, Anastasia Illarionova, Brit Mollenhauer, Christine Klein, Eva-Juliane Vollstedt, Franziska Hopfner, Günter Höglinger, Harutyun Madoev, Joanne Trinh, Johanna Junker, Katja Lohmann, Lara M. Lange, Manu Sharma, Sergio Groppa, Thomas Gasser, Zih-Hua Fang, Albert Akpalu, Georgia Xiromerisiou, Georgios Hadjigorgiou, Ioannis Dagklis, Ioannis Tarnanas, Leonidas Stefanis, Maria Stamelou, Efthymios Dadiotis, Alex Medina, Germaine Hiu-Fai Chan, Nancy Ip, Nelson Yuk-Fai Cheung, Phillip Chan, Xiaopu Zhou, Asha Kishore, Divya KP, Pramod Pal, Prashanth Lingappa Kukkle, Roopa Rajan, Rupam Borgohain, Mehri Salari, Andrea Quattrone, Enza Maria Valente, Lucilla Parnetti, Micol Avenali, Tommaso Schirinzi, Manabu Funayama, Nobutaka Hattori, Tomotaka Shiraishi, Altynay Karimova, Gulnaz Kaishibayeva, Cholpon Shambetova, Rejko Krüger, Ai Huey Tan, Azlina Ahmad-Annuar, Mohamed Ibrahim Norlinah, Nor Azian Abdul Murad, Norlinah Mohamed Ibrahim, Shahrul Azmin, Shen-Yang Lim, Wael Mohamed, Yi Wen Tay, Daniel Martinez-Ramirez, Mayela Rodriguez-Violante, Paula Reyes-Pérez, Bayasgalan Tserensodnom, Rajeev Ojha, Tim J. Anderson, Toni L. Pitcher, Arinola Sanyaolu, Njideka Okubadejo, Oluwadamilola Ojo, Jan O. Aasly, Lasse Pihlstrøm, Manuela Tan, Shoaib Ur-Rehman, Mario Cornejo-Olivas, Maria Leila Doquenia, Raymond Rosales, Angel Vinuela, Elena Iakovenko, Bashayer Al Mubarak, Muhammad Umair, Eng-King Tan, Jia Nee Foo, Ferzana Amod, Jonathan Carr, Soraya Bardien, Beomseok Jeon, Yun Joong Kim, Esther Cubo, Ignacio Alvarez, Janet Hoenicka, Katrin Beyer, Maria Teresa Periñan, Pau Pastor, Sarah El-Sadig, Christiane Zweier, Krack Paul, Chin-Hsien Lin, Hsiu-Chuan Wu, Pin-Jui Kung, Ruey-Meei Wu, Serena Wu, Yihru Wu, Rim Amouri, Samia Ben Sassi, A. Nazlı Başak, Gencer Genc, Özgür Öztop Çakmak, Sibel Ertan, Alastair Noyce, Alejandro Martínez-Carrasco, Anette Schrag, Anthony Schapira, Camille Carroll, Claire Bale, Donald Grosset, Eleanor J. Stafford, Henry Houlden, Huw R Morris, John Hardy, Kin Ying Mok, Mie Rizig, Nicholas Wood, Nigel Williams, Olaitan Okunoye, Patrick Alfryn Lewis, Rauan Kaiyrzhanov, Rimona Weil, Seth Love, Simon Stott, Simona Jasaitye, Sumit Dey, Vida Obese, Alberto Espay, Alyssa O'Grady, Andrew B Singleton, Andrew K. Sobering, Bernadette Siddiqi, Bradford Casey, Brian Fiske, Cabell Jonas, Carlos Cruchaga, Caroline B. Pantazis, Charisse Comart, Claire Wegel, Cornelis Blauwendraat, Dan Vitale, Deborah Hall, Dena Hernandez, Ejaz Shiamim, Ekemini Riley, Faraz Faghri, Geidy E. Serrano, Hampton Leonard, Hirotaka Iwaki, Honglei Chen, Ignacio F. Mata, Ignacio Juan Keller Sarmiento, Jared Williamson, Jonggeol Jeff Kim, Joseph Jankovic, Joshua Shulman, Justin C. Solle, Kaileigh Murphy, Karen Nuytemans, Karl Kieburtz, Katerina Markopoulou, Kenneth Marek, Kristin S. Levine, Lana M. Chahine, Laurel Screven, Lauren Ruffrage, Lisa Shulman, Luca Marsili, Maggie Kuhl, Marissa Dean, Mary B Makarious, Mathew Koretsky, Miguel Inca-Martinez, Mike A. Nalls, Naomi Louie, Niccolò Emanuele Mencacci, Roger Albin, Roy Alcalay, Ruth Walker, Sara Bandres-Ciga, Sohini Chowdhury, Sonya Dumanis, Steven Lubbe, Tao Xie, Tatiana Foroud, Thomas Beach, Todd Sherer, Yeajin Song, Duan Nguyen, Toan Nguyen, Masharip Atadzhanov Supplementary Material

Reporting Summary

## Data Availability

GP2 has partnered with the Accelerating Medicines Partnership - Parkinson’s Disease (AMP-PD; https://amp-pd.org) to share data generated by GP2, and in December 2021 the first GP2 genotyping data were released on the AMP-PD platform. As of 2023, the data consist of 14,902 samples (8190 PD cases), representing a broad range of diverse ancestries defined directly from the genotyping data from these cohorts. Genotyping and data QC is ongoing, and there will be regular data releases (2–4 times per year) as the project progresses. All contributing investigators have access to GP2 data, and external researchers can also gain access by following instructions on the GP2 website (https://gp2.org/applying-for-gp2-data-access-on-the-amp-pd-platform/). There are two tiers of data access. Tier 1 consists of summary statistics and any researcher can gain access by completing an online application. Tier 2 access includes deidentified, individual level genetic data, and to gain access researchers must sign a Data Usage Agreement co-signed by their institution.

## Code availability

Code used in GP2 core analyses and the custom clustering files are deposited in the GP2 GitHub repository (https://github.com/GP2code). Code created and used by the CIWG can be found on the “GP2-WorkingGroups/CD-Cohort-Integration” subpage of the GP2 GitHub repository (https://github.com/GP2code/GP2-WorkingGroups/tree/main/CD-Cohort-Integration). The Data and Code Dissemination Working Group (DCD-WG) at GP2 is committed to making these data, resources, and training materials accessible to the scientific community. The DCD-WG actively supports efforts to consolidate analysis scripts with the necessary analytical tools and decisions via GitHub (https://github.com/GP2code), consolidate training materials with several translations in collaboration with the Training and Networking Working Group (https://gp2.org/training/), and consolidate additional analytical pipelines on Terra (https://amp-pd.org/tools).
