# Supplementary material for: Defining the causes of sporadic Parkinson’s disease in the global Parkinson’s genetics program (GP2)
Source: NPJ Parkinsons Dis. 2023 Sep 12;9:131. doi: 10.1038/s41531-023-00533-w (PMC10497609; doi:10.1038/s41531-023-00533-w)
Supplement: Supplementary file 2 — Reporting Summary [file 41531_2023_533_MOESM2_ESM.pdf]

nature portfolio

Corresponding author(s): Prof Huw Morris (h.morris@ucl.ac.uk)

Last updated by author(s): Mar 22, 2023

Reporting Summary

Nature Portfolio wishes to improve the reproducibility of the work that we publish. This form provides structure for consistency and transparency in reporting. For further information on Nature Portfolio policies, see our Editorial Policies and the Editorial Policy Checklist.

Statistics

For all statistical analyses, confirm that the following items are present in the figure legend, table legend, main text, or Methods section.

n/a	Confirmed	
☒	☐	The exact sample size (*n*) for each experimental group/condition, given as a discrete number and unit of measurement
☒	☐	A statement on whether measurements were taken from distinct samples or whether the same sample was measured repeatedly
☒	☐	The statistical test(s) used AND whether they are one- or two-sided<br>*Only common tests should be described solely by name; describe more complex techniques in the Methods section.*
☒	☐	A description of all covariates tested
☒	☐	A description of any assumptions or corrections, such as tests of normality and adjustment for multiple comparisons
☒	☐	A full description of the statistical parameters including central tendency (e.g. means) or other basic estimates (e.g. regression coefficient) AND variation (e.g. standard deviation) or associated estimates of uncertainty (e.g. confidence intervals)
☒	☐	For null hypothesis testing, the test statistic (e.g. *F*, *t*, *r*) with confidence intervals, effect sizes, degrees of freedom and *P* value noted<br>*Give P values as exact values whenever suitable.*
☒	☐	For Bayesian analysis, information on the choice of priors and Markov chain Monte Carlo settings
☒	☐	For hierarchical and complex designs, identification of the appropriate level for tests and full reporting of outcomes
☒	☐	Estimates of effect sizes (e.g. Cohen's *d*, Pearson's *r*), indicating how they were calculated

Our web collection on statistics for biologists contains articles on many of the points above.

Software and code

Policy information about availability of computer code

Data collection	No software was used. Clinical data is uploaded to a secure cloud server by contributing cohorts. Samples from contributing cohorts are genotyped by GP2 on the Neuro Booster Array (https://github.com/GP2code/Neuro_Booster_Array).
Data analysis	This manuscript does not include results of data analysis. GP2 core analyses will be conducted by the Data Analysis Working Group using custom code, and the Data and Code Dissemination Working Group will make this code publicly available through the GP2 github repository (https://github.com/GP2code). The current manuscript provides an outline of the GP2 Complex Network Protocol and planned analyses. Data collection and analyses are ongoing and results will be reported in future manuscripts.

For manuscripts utilizing custom algorithms or software that are central to the research but not yet described in published literature, software must be made available to editors and reviewers. We strongly encourage code deposition in a community repository (e.g. GitHub). See the Nature Portfolio guidelines for submitting code & software for further information.

Data

Policy information about availability of data

All manuscripts must include a data availability statement. This statement should provide the following information, where applicable:

- Accession codes, unique identifiers, or web links for publicly available datasets
- A description of any restrictions on data availability
- For clinical datasets or third party data, please ensure that the statement adheres to our policy

GP2 has partnered with the Accelerating Medicines Partnership - Parkinson's Disease (AMP-PD; https://amp-pd.org) to share data generated by GP2, and in December 2021 the first GP2 genotyping data were released on the AMP-PD platform. As of 2023, the data consist of 14,902 samples (8,190 PD cases), representing a broad range of diverse ancestries defined directly from the genotyping data from these cohorts. Genotyping and data QC is ongoing, and there will be regular data releases (2-4 times per year) as the project progresses.

The Data and Code Dissemination Working Group (DCDWG) at GP2 is committed to making these data, resources, and training materials accessible to the scientific community. The DCDWG actively supports efforts to consolidate analysis scripts with the necessary analytical tools and decisions via GitHub (https://github.com/GP2code), consolidate training materials with several translations in collaboration with the Training and Networking Working Group (https://gp2.org/training/), and consolidate additional analytical pipelines on Terra (https://amp-pd.org/tools). All contributing investigators have access to GP2 data, and external researchers can also gain access by following instructions on the GP2 website (https://gp2.org/applying-for-gp2-data-access-on-the-amp-pd-platform/). There are two tiers of data access. Tier 1 consists of summary statistics and any researcher can gain access by completing an online application. Tier 2 access includes de-identified, individual level genetic data, and to gain access researchers must sign a Data Usage Agreement co-signed by their institution.

Human research participants

Policy information about studies involving human research participants and Sex and Gender in Research.

Reporting on sex and gender	No findings reported in this manuscript
Population characteristics	Recruitment to GP2 is ongoing. The target recruitment number is >150,000 samples contributed from different PD cohorts around the world. This will include a minimum of 50,000 individuals from ancestries currently underrepresented in PD research: including Black American, African, Middle Eastern, Central/ East/South Asian, Indian, Caribbean and Central/South American. The remaining participants are expected to be of European ancestry. Samples from both PD cases and controls are accepted from each cohort. Participants carrying known PD-associated genetic mutations are included. Cases diagnosed with Dementia with Lewy Bodies (DLB) and Parkinson's Disease Dementia (PDD) are also included. There is no age limit.
Recruitment	The GP2 Complex Network does not recruit patients directly. Existing studies across the globe contribute their samples/data to GP2, provided that written, informed consent for sample/data sharing was been obtained.
Ethics oversight	The GP2 Complex Network does not require ethical approval. All studies contributing samples/data to GP2 must have received ethical approval from their respective review boards prior to joining GP2.

Note that full information on the approval of the study protocol must also be provided in the manuscript.

Field-specific reporting

Please select the one below that is the best fit for your research. If you are not sure, read the appropriate sections before making your selection.

☒ Life sciences ☐ Behavioural & social sciences ☐ Ecological, evolutionary & environmental sciences

For a reference copy of the document with all sections, see nature.com/documents/nr-reporting-summary-flat.pdf

Life sciences study design

All studies must disclose on these points even when the disclosure is negative.

Sample size	A power calculation based on the 2019 Nalls et al., Parkinson's GWAS data indicates that inclusion of an additional ~99K cases would enable variants of smaller effect size that contribute to polygenic risk (p-value cut off: 1.35 x 10-3) to reach genome-wide significance. The 2019 GWAS included 37,688 cases. This is the basis for a target sample size of >150,000.
Data exclusions	Data collection and analyses are ongoing, results are not reported in the current manuscript.
Replication	Data collection and analyses are ongoing, results are not reported in the current manuscript.
Randomization	Not relevant, samples are categorised as case or control according to clinical diagnosis.
Blinding	Not relevant, case/control status needs to be known for GWAS and other genotype-phenotype analyses.

Reporting for specific materials, systems and methods

We require information from authors about some types of materials, experimental systems and methods used in many studies. Here, indicate whether each material, system or method listed is relevant to your study. If you are not sure if a list item applies to your research, read the appropriate section before selecting a response.

Materials & experimental systems

n/a	Involved in the study
☒	☐ Antibodies
☒	☐ Eukaryotic cell lines
☒	☐ Palaeontology and archaeology
☒	☐ Animals and other organisms
☒	☐ Clinical data
☒	☐ Dual use research of concern

Methods

n/a	Involved in the study
☒	☐ ChIP-seq
☒	☐ Flow cytometry
☒	☐ MRI-based neuroimaging
